# Circulating Neutrophil Profiles Undergo a Dynamic Shift during Metabolic Dysfunction-Associated Steatohepatitis (MASH) Progression

**DOI:** 10.3390/biomedicines12051105

**Published:** 2024-05-16

**Authors:** Ana C. Maretti-Mira, Matthew P. Salomon, Shefali Chopra, Liyun Yuan, Lucy Golden-Mason

**Affiliations:** 1 USC Research Center for Liver Diseases, Division of Gastrointestinal and Liver Diseases, Department of Medicine, Keck School of Medicine, University of Southern California, Los Angeles, CA 90033, USA; msalomon@usc.edu (M.P.S.); lyuan@med.usc.edu (L.Y.); lucy.golden@med.usc.edu (L.G.-M.); 2 Department of Pathology, Keck School of Medicine, University of Southern California, Los Angeles, CA 90033, USA; shefali.chopra@med.usc.edu

**Keywords:** metabolic dysfunction-associated steatotic liver disease (MASLD), innate immunity, transcriptomics, inflammation, metabolic dysfunction-associated steatohepatitis (MASH), neutrophils

## Abstract

Neutrophils play a crucial role in host defense against infection. Aberrant neutrophil activation may induce tissue damage via sterile inflammation. Neutrophil accumulation has been identified as a feature of the inflammatory response observed in metabolic dysfunction-associated steatohepatitis (MASH) and has been associated with liver fibrosis and cirrhosis. Here, we performed the transcriptomic analysis of circulating neutrophils from mild and advanced MASH patients to identify the potential mechanism behind neutrophil contribution to MASH progression. Our findings demonstrated that circulating neutrophils from mild and advanced MASH display an increased activated transcriptional program, with the expression of pro-inflammatory factors and an amplified lifespan compared to cells from non-diseased controls. Our results also suggest that MASH progression is associated with a dynamic shift in the profile of circulating neutrophils. In the early stages of MASH, mature neutrophils predominate in the bloodstream. As hepatic inflammation and fibrosis progress, the premature release of immature neutrophils into the circulation occurs. These immature neutrophils exhibit a pro-inflammatory profile that may exacerbate inflammation and promote fibrosis in MASH.

## 1. Introduction

Neutrophils, the most abundant white blood cells in the circulation, are the first innate immune cell type to reach sites of injury or inflammation [1]. Upon recruitment to the inflammatory site, neutrophils perform their effector functions, which include the phagocytosis and destruction of microorganisms, the generation of reactive oxygen species (ROS) and serine proteases, and the production of pro-inflammatory cytokines and chemokines to amplify the innate and adaptive immune responses [2]. However, their highly destructive capacity can potentially damage healthy tissues even in the absence of infection [3]. Neutrophils are short-lived and have a circulating half-life of approximately 1.5 h in mice and 8 h in humans [4]. Once they infiltrate tissue, neutrophils can be activated by various triggers, including inflammatory cytokines and growth factors, which can significantly increase their lifespan [5]. Neutrophil apoptosis ensures their removal by Kupffer cells in the liver and serves as a critical control point in resolving inflammation. The dysregulation of neutrophil death or delayed apoptosis is thought to contribute to a wide range of inflammatory pathologies. Pro-survival and pro-apoptosis signals from the inflammatory environment can disturb the execution of constitutive death programs, thereby affecting the fate of neutrophils and the outcome of the inflammatory response [6].

Several lines of evidence suggest that neutrophils participate in the progression of metabolic dysfunction-associated steatotic liver disease (MASLD), previously known as nonalcoholic liver disease (NAFLD) [7,8]. Predicted to become the leading indication for liver transplantation [9], MASLD is the most common chronic liver disease in modern society [10]. It encompasses a spectrum of liver pathologies ranging from bland steatosis to metabolic dysfunction-associated steatohepatitis (MASH), formerly known as nonalcoholic steatohepatitis (NASH). MASH is characterized by liver injury, inflammation, and fibrosis, and in severe cases, it can lead to cirrhosis, liver failure, and hepatocellular carcinoma. MAFLD development is driven by inflammation, and innate immunity has been identified as a key contributor to disease progression [11].

Patients with MASH exhibit a significant elevation in the neutrophil-to-lymphocyte ratio (NLR), a potential non-invasive biomarker for the disease. This increase in NLR correlates positively with the histological scores of hepatocyte ballooning, lobular inflammation, and fibrosis in MASH patients [12]. Several neutrophil-derived granular proteins, including neutrophil elastase (NE), proteinase 3 (PR3), and neutrophil gelatinase-related lipocalin (NGAL/LCN2) [13,14], are also elevated in the plasma of MASH subjects. Myeloperoxidase (MPO), a powerful pro-oxidative and proinflammatory protein mainly released by activated neutrophils, is also significantly increased in the serum of MASH patients [15]. Additionally, hepatic neutrophil accumulation is a hallmark of MASH, and the hepatic proportion of neutrophils strongly correlates with the plasma levels of alanine transaminase (ALT), aspartate transaminase (AST), and gamma-glutamyl transferase (GGT), markers of liver damage [16]. While these data support that neutrophils may contribute to hepatocellular injury and fibrosis during MAFLD/MASH progression, the mechanisms underlying this process are poorly understood. A recent study suggests that peripheral neutrophils may already be activated before trafficking to the liver, potentially prolonging their survival [15]. However, a better understanding of the neutrophil contribution to MASH is essential before assessing whether their modulation represents an effective therapeutic approach for preventing disease progression. In the present study, we conducted a bulk transcriptional analysis of circulating neutrophils from biopsy-proven mild and advanced MASH patients and found that neutrophil profiles are profoundly altered during disease progression.

## 2. Materials and Methods

### 2.1. Patient Selection

Seven obese patients (BMI ≥ 30 kg/m^2^) with liver biopsy-confirmed MASH diagnosis within the previous six months were included in this study. To verify the clinical diagnosis, cases were retrospectively reviewed by a single pathologist (S.C). Histologic activity was assessed using the NAFLD activity score (NAS), and fibrosis (F) was evaluated using NASH CRN classification [17]. Subjects were classified as mild (NAS ≤ 3, F0–1, n = 3) or advanced (NAS ≥ 5 and/or F ≥ 4, n = 4) MASH. Five non-diseased lean donors with no history of liver disease or diabetes served as control subjects. This study was approved by the Institutional Review Board University of Southern California, and blood collection was conducted with the agreement and written consent of each participant.

### 2.2. Sample Collection and Isolation of Neutrophils

Human neutrophils were obtained from the subject’s blood collected in Vacutainer EDTA tubes (BD Biosciences, Franklin Lakes, NJ, USA). To avoid neutrophil activation, cells were purified via negative selection using the MACSxpress^®^ Whole Blood Neutrophil Isolation Kit, Human (Miltenyi Biotech, Gaithersburg, MD, USA) following the manufacturer’s protocol. The purity of isolated neutrophils was assessed using flow cytometry (CD15^+^CD16^+^CD14^−^CD193^−^). Samples included in this study displayed a purity average of 95%. Cell pellets were stored at −80 °C in 700 µL of RLT/BME Buffer (Qiagen, Germantown, MD, USA) for subsequent RNA extraction and processing.

### 2.3. Bulk RNA-Seq

Library preparation and sequencing: RNA was isolated using RNeasy Mini kit (Qiagen, Germantown, MD, USA) and RNA integrity was analyzed by 2100 Expert Bioanalyzer System (Agilent Technologies, Carpinteria, CA, USA), using the Agilent RNA 6000 Pico Kit (Agilent Technologies, Carpinteria, CA, USA). Libraries were simultaneously prepared from extracted total RNA using the Illumina Truseq Stranded mRNA library preparation kit according to the manufacturer’s protocol (Illumina, San Diego, CA, USA). Prepared libraries were sequenced on the Illumina Nextseq500 at 30 million reads per sample at 2 × 75 cycles. Libraries were prepared and sequenced by the University of Southern California (USC) Molecular Genomics Core, Los Angeles, CA, USA.

### 2.4. Bioinformatics and Statistical Analyses

#### 2.4.1. Raw Data Processing

The raw sequencing reads were first checked for overall quality and adapter contamination using FastQC (version 0.11.9) [18] and trimmed using TrimGalore (version 0.6.6) [19] before downstream analysis. Reads were then mapped to the GENCODE version 35 human genome reference using the STAR aligner (version 2.7.6a) [20].

#### 2.4.2. Statistical Analyses

Significantly differentially expressed genes were identified using the Bioconductor (version 3.12) package DESeq2 (version 1.30.0) [21] with a model that accounted for the effects of sex (i.e., ~sex + group) and a significance threshold of an adjusted *p*-value of <0.1. A likelihood ratio test was used to detect significantly differentially expressed genes across disease states and the function degPatterns from the Bioconductor package DEGreport (version 1.25.1) [22] was used to identify groups of genes with common expression patterns. Differentially expressed genes were visualized as volcano plots using functions from the Bioconductor package EnhancedVolcano (version 1.8) [23]. Only genes that were statistically significant concerning differential expression, as described in raw data processing above, were used in the subsequent analysis. Plots were created using GraphPad Prism version 10.

#### 2.4.3. Biological Interpretation

Ingenuity Pathway Analysis (IPA) software (version 01-22-01), was used to determine the canonical pathways and biological processes altered in the different groups [24]. The g:GOSt tool from the g:Profiler platform was used to identify the enrichment of biological processes using the differentially expressed genes [25]. GeneMANIA was used to evaluate the strength of the networks, with the following criteria: (i) network weighting method—biological process-based (Gene Ontology); (ii) up to 20 additional genes; (iii) up to 10 attributes and considered (iv) co-expression, (v) co-localization, (vi) predicted interaction, and (vii) genetic and physical interactions; and (viii) common pathways [26].

## 3. Results

### 3.1. Transcriptomic Profiling of Circulating Neutrophils in MASH Patients

**Figure 1:**
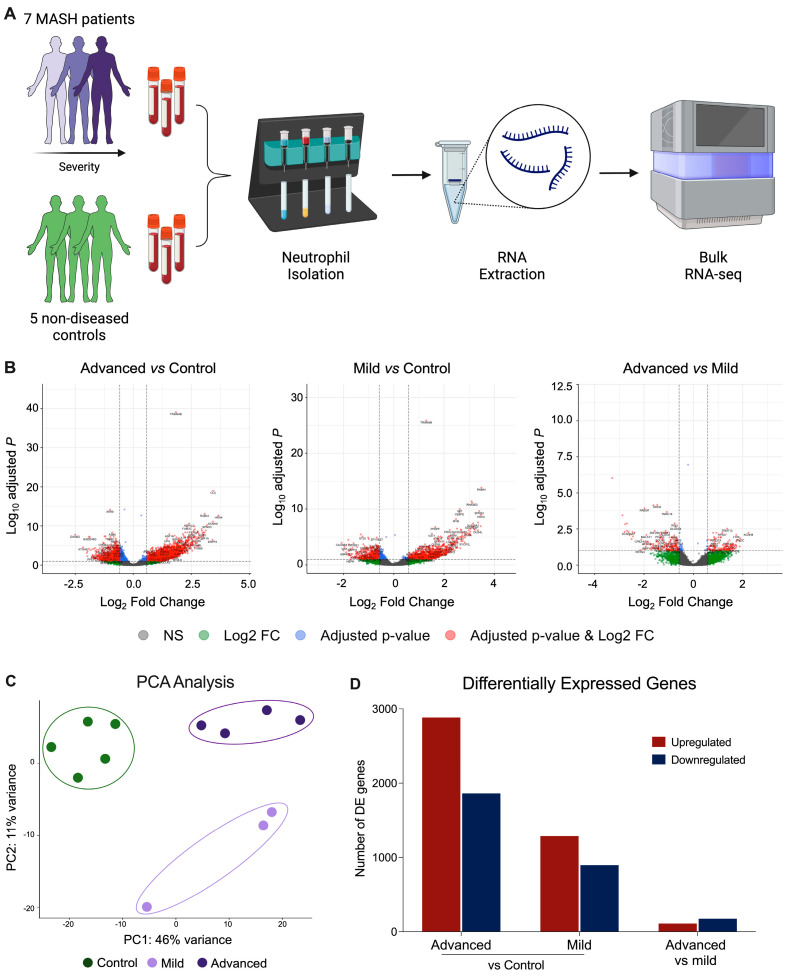
Transcriptomic characterization of neutrophils circulating in MASH patients. (**A**) Overall experimental design applied to this study. (**B**) Volcano plots display the distribution of genes expressed by MASH subjects compared to non-diseased control individuals and between advanced and mild MASH. (**C**) PCA plot illustrating the relationship of all samples based on dynamic gene expression of all genes comparing mild and advanced MASH with control groups. (**D**) Number of significantly upregulated (red) and downregulated (blue) genes (FC > 1.2, adj. *p* value < 0.1) comparing MASH and control samples, and advanced vs. mild MASH).

Patients with different stages of MASH were recruited to this study and grouped according to their biopsy NAFLD activity score in mild (Mild-MASH, NAS ≤ 3 or F0–1) or advanced (ADV-MASH, NAS ≥ 5 or F3 or greater) MASH (Table 1). Total neutrophils were isolated from peripheral blood by negative selection to avoid neutrophil activation, and transcriptional datasets were obtained via bulk RNA sequencing (RNA-seq) (Figure 1A).

The global transcriptional analyses of purified circulating neutrophil populations demonstrated a highly activated transcriptional program for both mild and advanced cases when compared to control subjects (Figure 1B). Principal component analysis (PCA) revealed a clear distinction between biological replicates, with the well-defined separation of the control, mild, and advanced MASH groups (Figure 1C). Compared to non-diseased control subjects, neutrophils from ADV-MASH patients they displayed 4745 differentially expressed genes (DEGs), with 2883 upregulated and 1862 downregulated genes. Neutrophils from Mild-MASH patients displayed 2185 DEGs, with 1288 upregulated and 897 downregulated genes (Figure 1D). In a direct comparison, neutrophils from mild and advanced cases showed 284 DEGs, with 110 upregulated and 174 downregulated genes.

**Figure 2:**
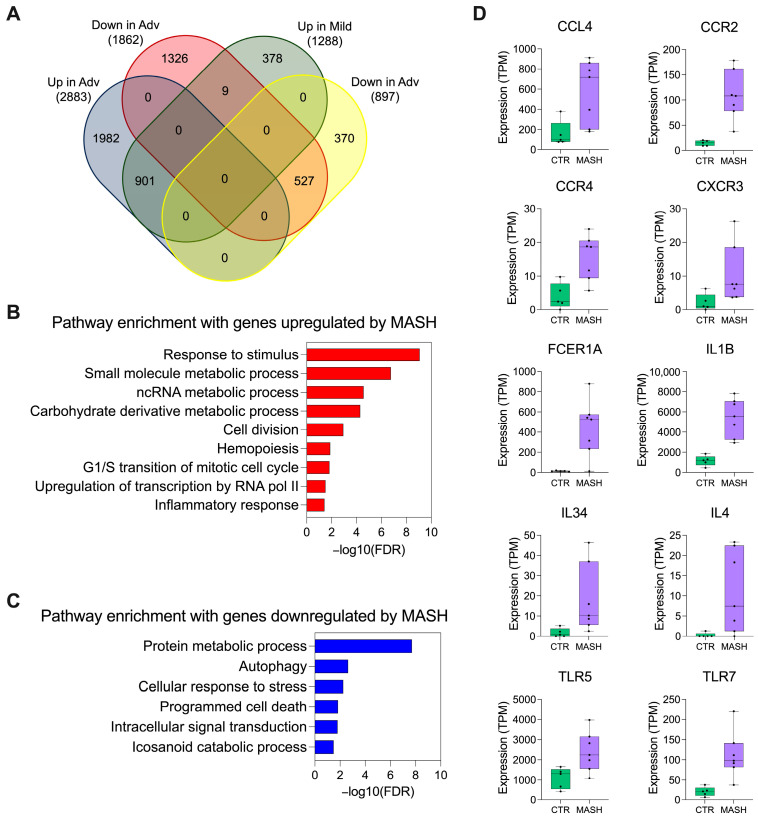
Genes commonly affected by MASH in circulating neutrophils. (**A**) Venn diagram illustrating the unique and common differentially expressed genes in neutrophils from mild and advanced MASH cases. Genes were grouped according to regulation direction. (**B**) Functional enrichment analysis of genes commonly upregulated in mild and advanced MASH. (**C**) Functional enrichment analysis of genes commonly downregulated in mild and advanced MASH. (**D**) Differentially expressed genes commonly upregulated in neutrophils from MASH patients with relevant immunological function for MAFLD progression.

We found that the 901 genes commonly upregulated in the MASH subjects (Figure 2A) enriched pathways were related to RNA metabolic processing, cell division, and immune response (Figure 2B), while the 527 genes commonly downregulated in advanced and mild groups were involved in protein metabolism, autophagy, response to cellular stress, and apoptosis (Figure 2C). Among the genes significantly upregulated in neutrophils from MASH patients that participate in the inflammatory response, we found CCL4, CCR2, CCR4, CXCR3, FCER1A, IL1B, IL34, IL4, TLR5, and TLR7 (Figure 2D). The genes ZNF865, MFSD14CP, LOC128125822, LRFN1, TNRC18, RAB5IF, MAP1S, GNG5, and SCAF1 were downregulated with the progression of the disease (i.e., up in mild and down in advanced). Most of these genes participate in activities related to mitochondria respiration (Figure S1A,B). These results suggest that neutrophil transcriptomic profiles change during MAFLD progression, and the advanced stages of MASH display the most robust differences.

### 3.2. Severe MASH Stimulates Premature Neutrophil Release into the Bloodstream

**Figure 3:**
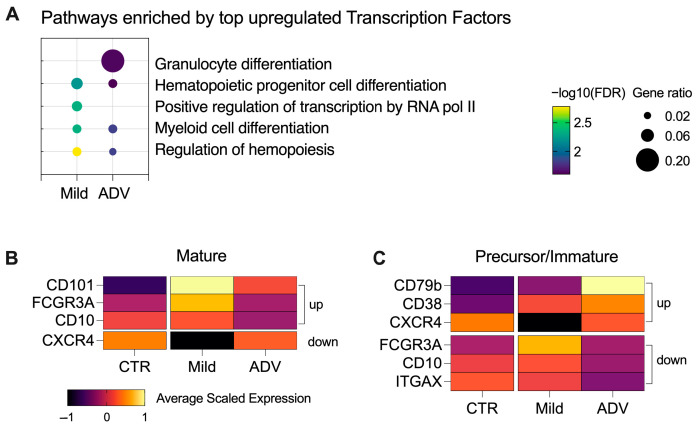
Maturation profile of circulating neutrophils changes during MASH progression. (**A**) Functional enrichment pathway analysis of top transcription factors upregulated in neutrophils from mild and advanced MASH patients. (**B**) Heatmap showing the average scaled gene expression of biomarkers expressed by mature neutrophils. The right-side legend indicates the expected direction of gene regulation (up or down) in classic mature neutrophils. (**C**) Heatmap showing the average scaled gene expression of biomarkers expressed by precursor and/or immature neutrophils. The right-side legend indicates the expected direction of gene regulation (up or down) in precursor/immature neutrophils.

To elucidate the homeostatic disparities between circulating neutrophils in mild and advanced MASH cases, we analyzed their top-expressed transcription factors and discovered a significant enrichment in pathways associated with cell differentiation (Figure 3A). Subsequently, we assessed the markers for precursor, immature, and mature neutrophils (Figure 3B) [27,28]. We observed that neutrophils from Mild-MASH patients displayed a high expression of CDC101, FCGR3A, and CD10, and a low expression of CXCR4. This pattern suggests that there is a prevalence of mature neutrophils circulating in individuals at early stages of MASH. Interestingly, we found that the neutrophils from ADV-MASH patients exhibited a strong expression of CD79B, CD38, and CXCR4 and a downregulation of FCGR3A, CD10, and ITGAX. This gene expression pattern is found in neutrophil precursors and suggests the advancement of hepatic inflammation and fibrosis may induce the premature release of neutrophils into the bloodstream of individuals in later stages of MASH.

When directly comparing the transcriptomes of Mild- and AVD-MASH neutrophils (advanced × mild DEGs), we found that neutrophils from Mild-MASH cases expressed high levels of the pro-fibrotic factor TGFB1, the microfibrillar collagen COL6A3 and the pro-fibrotic metalloproteinases MMP25 and ADAMTSL4. The genes upregulated in neutrophils from early MASH-enriched pathways related to inflammatory response, cell migration, and degranulation. These findings suggest that in addition to degranulation and NET formation, mature neutrophils may also contribute to fibrosis progression in the early stages of MASH (Figure S2A,B). On the other hand, the genes upregulated in ADV-MASH neutrophils enriched pathways related to RNA translation, hepatic steatosis, and apoptosis. Of note, these cells also expressed higher levels of HLA class II (HLA-DOA, HLA-DMA, HLA-DOB, HLA-DMB,) FC receptors (FCRLA and FCRL1), MSR1 (scavenger receptor), and KLRF1 (C-type lectin-like receptor), suggesting that circulating neutrophils in severe MASH may have the increased recognition and clearance of apoptotic cells and pathogens and antigen-presenting functions.

### 3.3. Circulating Neutrophils Exhibit Pro-Inflammatory and Pro-Fibrotic Traits in MASH

**Figure 4:**
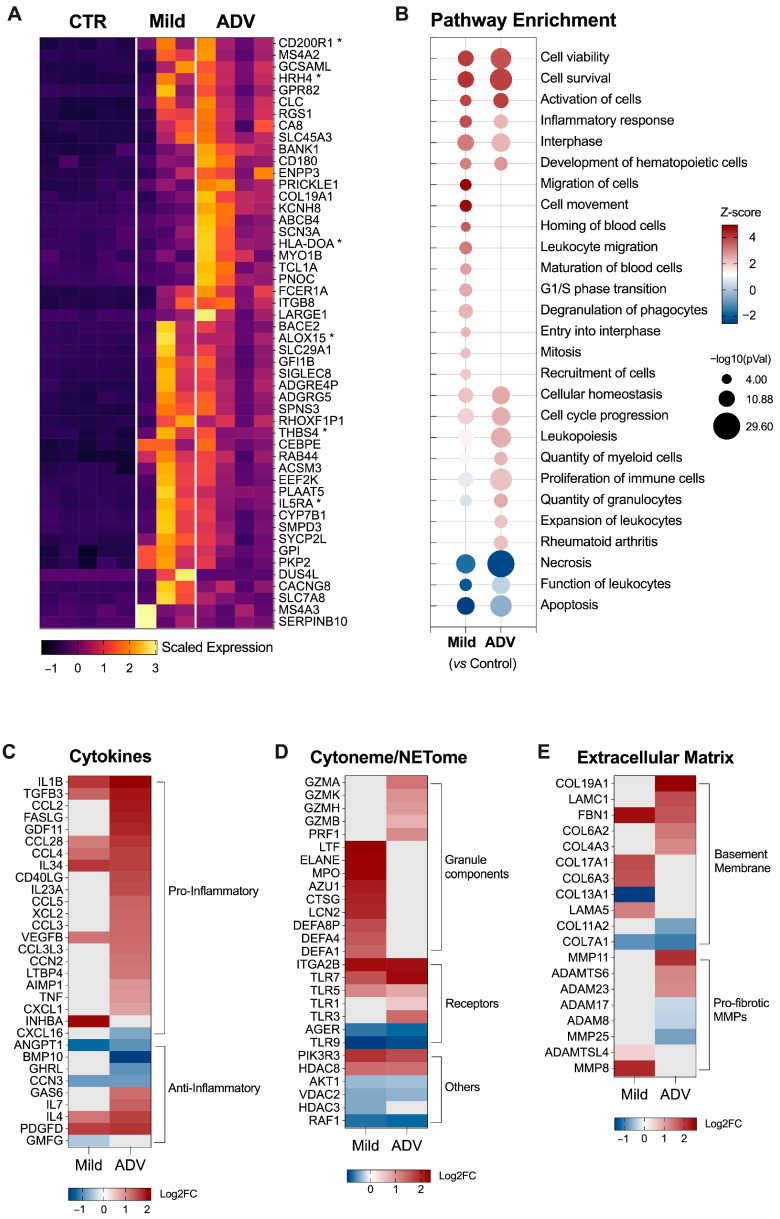
Circulating neutrophils express pro-inflammatory and pro-fibrotic genes in MASH patients. (**A**) Heatmap showing the top 50 upregulated genes in neutrophils from mild and advanced MASH. * Genes with relevant immunological function to MAFLD progression or for neutrophil-mediated inflammatory response. Values are shown as scaled gene expression. (**B**) Pathways enriched by genes differentially expressed in mild and advanced MASH compared to control. (**C**) Heatmap displaying cytokines expressed by MASH-derived neutrophils, grouped as pro- and anti-inflammatory. (**D**) Heatmap showing the expression of genes encoding granules proteins involved in degranulation (cytoneme) and/or neutrophils extracellular trap formation (NETome). (**E**) Heatmap displaying genes encoding basement membrane compounds and pro-fibrotic metalloproteinases (MMPs). Log2FC, log2-foldchange based on control samples.

Among the top 50 genes upregulated in the MASH cases, we found IL15RA, CD200R1, ALOX15, HLA-DOA, HRH4, and THBS4, which are related to inflammation and fibrosis (Figure 4A). Using IPA™ analysis to evaluate the DEGs from mild and advanced groups, we identified several biological processes with the potential to impact MASH progression (Figure 4B). We observed that neutrophils from Mild-MASH and ADV-MASH showed the upregulation of biological processes involved in cell viability and activation and the inflammatory response. Neutrophils from Mild-MASH also demonstrated upregulation of pathways related to cell migration, maturation, and degranulation, while cells from ADV-MASH showed the upregulation of cell proliferation.

Neutrophils from ADV-MASH showed a remarkable upregulation of pro-inflammatory cytokines, including several chemoattractants, namely the pro-fibrotic factors TGFB3, LTBP4, and IL34 (Figure 4C). Neutrophils from Mild-MASH cases displayed a high expression of genes involved in degranulation and neutrophil extracellular trap formation (NETosis), with a strong upregulation of ELAN, MPO, and AZU1 (Figure 4D). Although neutrophils from ADV-MASH did not show the significant upregulation of antimicrobial peptide genes, they demonstrated the upregulation of genes encoding granzymes and perforin, suggesting that these cells may play a role in polymorphonuclear cell-mediated antibody-dependent cellular cytotoxicity in severe MASH. Neutrophils from both groups express fibrillar and non-fibrillar collagens, laminin and fibrillin 1, as well as pro-fibrotic metalloproteinases, suggesting that neutrophils may contribute to fibrosis progression in MASH (Figure 4E).

### 3.4. MASH-Driven Alterations in Neutrophil Profiles Correlate with Disease Progression

**Figure 5:**
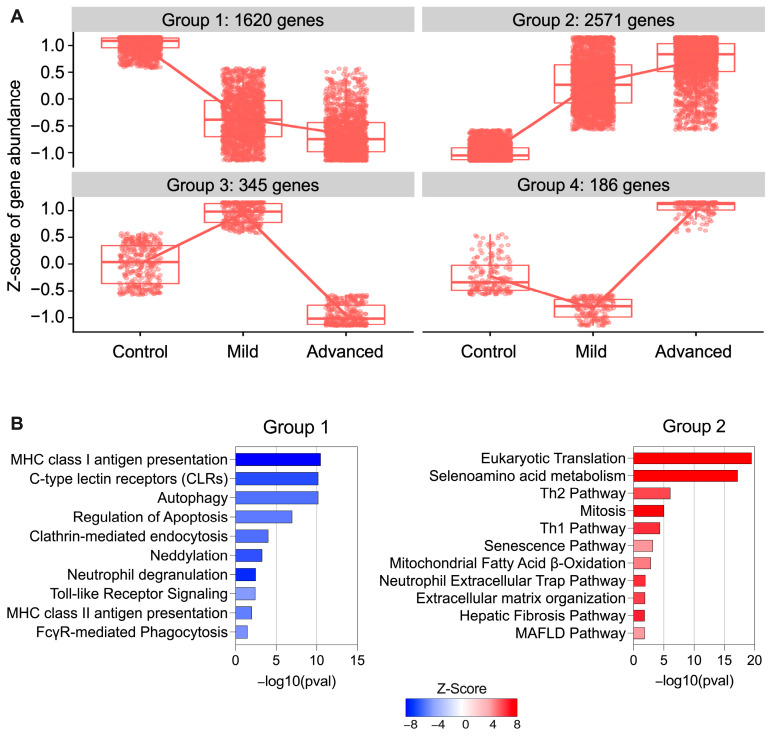
MASH progression directly impacts the circulating neutrophil transcriptome. (**A**) Boxplot showing the likelihood ratio test (LRT) results, where we identified the gene clusters that follow a specific pattern during the evolution of the disease. (**B**) Functional enrichment pathway analysis of genes identified in groups 1 and 2. Pathways were ranked according to −log10(pval) and colored according to the Z-score of activation.

We then hypothesized that disease evolution affects the gene expression of circulating neutrophils (CTR < Mild < ADV and CTR > Mild > ADV). To test this hypothesis, we used the likelihood ratio test (LRT) and found that 4722 genes followed a specific pattern across samples that could correlate with MASH progression. We observed that most of the genes (55%) gradually upregulated their expression during MASH progression (group 2, Figure 5A) and that 34% of the genes exhibited lower expression with disease development (group 1, Figure 5A). Only a small group of genes diverged between Mild and ADV stages (groups 3 and 4, Figure 5A).

After identifying the group of genes that were modulated by disease progression, we analyzed their functional significance (Figure 5B). Pathways related to antigen presentation, pathogen pattern recognition, autophagy, apoptosis, endocytosis, and degranulation are possibly inhibited with MASH progression. However, these cells are likely to increase the RNA translation, NET formation, and activation of adaptive immune response during MASH development. These findings suggest that neutrophils undergo a change in their immunological and homeostatic behaviors during disease evolution, becoming less prone to regular degranulation and favoring the establishment of inflammation and fibrosis, which may ultimately contribute to MASH progression.

## 4. Discussion

MASH, the more severe form of MAFLD, is characterized by inflammation, hepatic damage, and fibrosis [29]. The innate inflammatory response is a key factor in triggering MASH [11]. Accumulating evidence supports that neutrophils may be a suitable target for treating MASH [7]. However, neutrophil participation in disease progression is not clear, and a better understanding of the molecular mechanisms underlying neutrophil function is essential for developing new therapies for MAFLD. To address this knowledge gap, we evaluated the transcriptome of circulating neutrophils isolated from the peripheral blood of MASH patients at different stages of the disease, which were classified as Mild-MASH (i.e., low inflammation scores and absent/low fibrosis) and ADV-MASH (i.e., high inflammation and fibrosis scores). Our findings suggest that neutrophils may play a more complex and multifaceted role in MASH than previously thought.

As evidenced by the large number of differentially expressed genes detected in our study, circulating neutrophils are activated in MASH. Among the top upregulated genes in neutrophils from mild and advanced MASH, we found genes that have already been associated with MAFLD progression or with pro-inflammatory neutrophils. Of note, we found CCL4, IL1B, IL34, IL15R, Alox5, CD200R1, THBS4, and HRH4. CCL4 (MIP-1β) is a proinflammatory chemokine that recruits natural killer cells, monocytes, and neutrophils to inflammatory sites [30,31]. IL1B and IL34 are both pro-inflammatory cytokines that promote macrophage recruitment and retention and hepatic stellate cell activation [32,33,34]. IL15 signaling is associated with high-fat diet-induced lipid accumulation and inflammation in the liver and with neutrophil phagocytosis competence [35,36]. Alox15 signaling is involved in liver injury pathogenesis in experimental models of hyperlipidemia-derived MAFLD and enhances neutrophil recruitment and inflammatory response [37,38,39]. The CD200R1:CD200 axis is reported as important for reactive oxygen species production (ROS) by neutrophils [40]. Thrombospondin-4 (THBS4) signaling facilitates macrophage differentiation into a pro-inflammatory phenotype [41]. The histamine 4 receptor (HRH4) is a potent inhibitor of adhesion-dependent neutrophil degranulation [42]. Regarding the functional pathways analysis of the differentially expressed genes in mild and advanced MASH neutrophils, we found an enrichment of pathways related to increased cell survival, with the marked inhibition of apoptosis. In normal physiological conditions, neutrophils are short-lived cells. However, our findings support that in MASH, circulating neutrophils display a longer lifespan and pro-inflammatory features that may promote tissue injury upon neutrophil recruitment to the liver, ultimately contributing to MASH progression.

Although neutrophils from mild and advanced MASH cases share several similarities, these cells also display different immunological traits according to disease stage. In Mild-MASH, we observed neutrophils with the increased expression of markers that indicate the prevalence of a mature neutrophil population. The maturation status of neutrophils has been linked to granule-associated functionality [43]. Our data confirmed that these cells expressed high levels of myeloperoxidase (MPO), azurophil (AZU1), elastase (ELANE), cathepsin G (CTSG), and lipocalin-2 (LCN2), important proteins that constitute the prepackaged neutrophil granules. Moreover, these cells also displayed an enrichment of migratory/homing and degranulation activities. Together with the inhibition of apoptosis shown by the cells from this group, our data suggest that, in the initial stages of MASH, circulating neutrophils are predominantly mature with a high probability of homing to hepatic inflamed tissue. Once infiltrating the liver, these cells might live longer and be more prone to degranulate, which would consequently aggravate MASH-derived hepatic injuries.

On the other hand, circulating neutrophils from patients with advanced MASH expressed high levels of precursors and immature neutrophil markers. This neutrophil pattern is often observed during severe infection or systemic inflammation conditions due to a process known as emergency granulopoiesis. This adaptive mechanism enhances neutrophil output from the bone marrow to compensate for the increased consumption of these cells during the innate immune response [44]. Prematurely released neutrophils might also represent low-density neutrophils (LDNs), a neutrophil subset that display proinflammatory, degranulation, and immunosuppressive features [45]. LDNs have been reported in obesity and other hepatic diseases [46,47]. We examined the known markers of LDNs suggested by various studies, including CD33, CD66b, CD36, CD41, CD61, CD226, ORL1, CD11b, and CD15, in both Mild and ADV samples [47,48]. However, our analysis did not reveal a significant presence of LDN markers in either group. While we acknowledge the possibility of LDNs being present, our dataset does not provide evidence to support this observation.

Immature neutrophils circulating in advanced MASH patients also expressed an array of pro-inflammatory chemokines that can aggravate and perpetuate liver inflammation, such as CCL2, CCL3, CCL5, CXCL1, and XCL2. CCL2, CCL3, and CCL5 facilitate the recruitment of monocytes and macrophages, important cells implicated in MAFLD pathogenesis [35,49,50]. CXCL1 recruits neutrophils [39], and XCL2 recruits XCR1^+^ dendritic cells [51]. Furthermore, these cells also express TNF, a pro-inflammatory cytokine that can increase the expression of key molecules involved in lipid metabolism, inflammation, and fibrosis in the liver [52]. Another interesting feature of circulating neutrophils in advanced MASH is their increased expression of HLA class II, which can be a result of the typical systemic inflammation associated with MASH [53,54], and the increased expression of granzymes and perforin, suggesting they have acquired cytotoxic traits. Thus, our findings suggest that the later stages of MASH are marked by the premature release of immature neutrophils that, upon infiltrating the liver, could exacerbate the tissue inflammation by recruiting other inflammatory cells, activating innate and adaptive immune cells via antigen presentation, and enhancing liver injury via cell-mediated cytotoxic reactions.

Overall, our findings shed new light on the immunological mechanisms driving neutrophil involvement in MASH progression and provide valuable insights for developing therapeutic strategies targeting these cells to prevent fibrosis and enhance treatment outcomes. While our findings support the active role of neutrophils in MASH progression, we cannot rule out the influence of several factors related to the underlying MASH pathophysiology that might have a synergistic impact on neutrophil behavior, such as progressive inflammation, oxidative stress, and liver injury and age, genetic background, and other metabolic disorders associated with the disease [55,56,57,58,59]. A limitation of our study lies in its small sample size and reliance on RNA-Seq alone to assess circulating neutrophils. However, the disease stage was confirmed by histological analysis and, despite the small sample size, our gene expression analysis provides valuable data to help us understand the role played by neutrophils in MASH progression. Our dataset provides a resource to inform other researchers and aid in the design of hypothesis-driven studies. Future functional and protein analyses would be beneficial to validate this functional shift.

## Figures and Tables

**Table 1:** Clinical characteristics of patients included in this study.

Clinical Parameters	Control (*n* = 5)	Mild (*n* = 3)	Adv (*n* = 4)
Age, years (mean, range)	40 (35–56)	49 (41–55)	54 (30–71)
Sex, female/male (*n*)	3/2	1/2	3/1
BMI, kgm^−2^ (mean, range)	25 (23–27)	32 (29–33)	34 (31–37)
NAS score (mean, range)	NA	2 (2–3)	4 (2–5)
Fibrosis score (mean, range)	NA	0 (0–1)	3 (2–4)
ALT, U/L (mean, range)	NA	65 (32–98)	82 (38–160)
AST, U/L (mean, range)	NA	30 (19–41)	37 (38–107)
Diabetes (*n*)	0	0	2

ALT, alanine aminotransferase; AST, aspartate aminotransferase; U/L, units per liter; NA, not applicable.

## Data Availability

The datasets generated for this study can be found in the Gene Expression Omnibus (GEO) at https://www.ncbi.nlm.nih.gov/geo/query/acc.cgi?acc=GSE247467.

## Supplementary Materials

The following supporting information can be downloaded at: https://www.mdpi.com/article/10.3390/biomedicines12051105/s1. Figure S1: Identification of genes that shift expression in neutrophils from mild to advanced MASH patients; Figure S2: Direct comparison between advanced and mild MASH.
