# Supplementary material for: Circulating Neutrophil Profiles Undergo a Dynamic Shift during Metabolic Dysfunction-Associated Steatohepatitis (MASH) Progression
Source: Biomedicines. 2024 May 16;12(5):1105. doi: 10.3390/biomedicines12051105 (PMC11117983; doi:10.3390/biomedicines12051105)
Supplement: Supplementary file 1 [file biomedicines-12-01105-s001.zip › biomedicines-3003589-supplementary.pdf]

Article

Circulating Neutrophil Profiles Undergo a Dynamic Shift during Metabolic Dysfunction-Associated Steatohepatitis (MASH) Progression

Ana C. Maretti-Mira [1,*], Matthew P. Salomon [1], Shefali Chopra [2], Liyun Yuan [1] and Lucy Golden-Mason [1]

[1] USC Research Center for Liver Diseases, Division of Gastrointestinal and Liver Diseases, Department of Medicine, Keck School of Medicine, University of Southern California, Los Angeles, CA 90033, USA; msalomon@usc.edu (M.P.S.); lyuan@med.usc.edu (L.Y.); lucy.golden@med.usc.edu (L.G.-M.)
[2] Department of Pathology, Keck School of Medicine, University of Southern California, Los Angeles, CA 90033, USA; shefali.chopra@med.usc.edu
* Correspondence: marettim@usc.edu

Supplementary Figures

Content List:

Supplemental Figure 1

Supplemental Figure 2

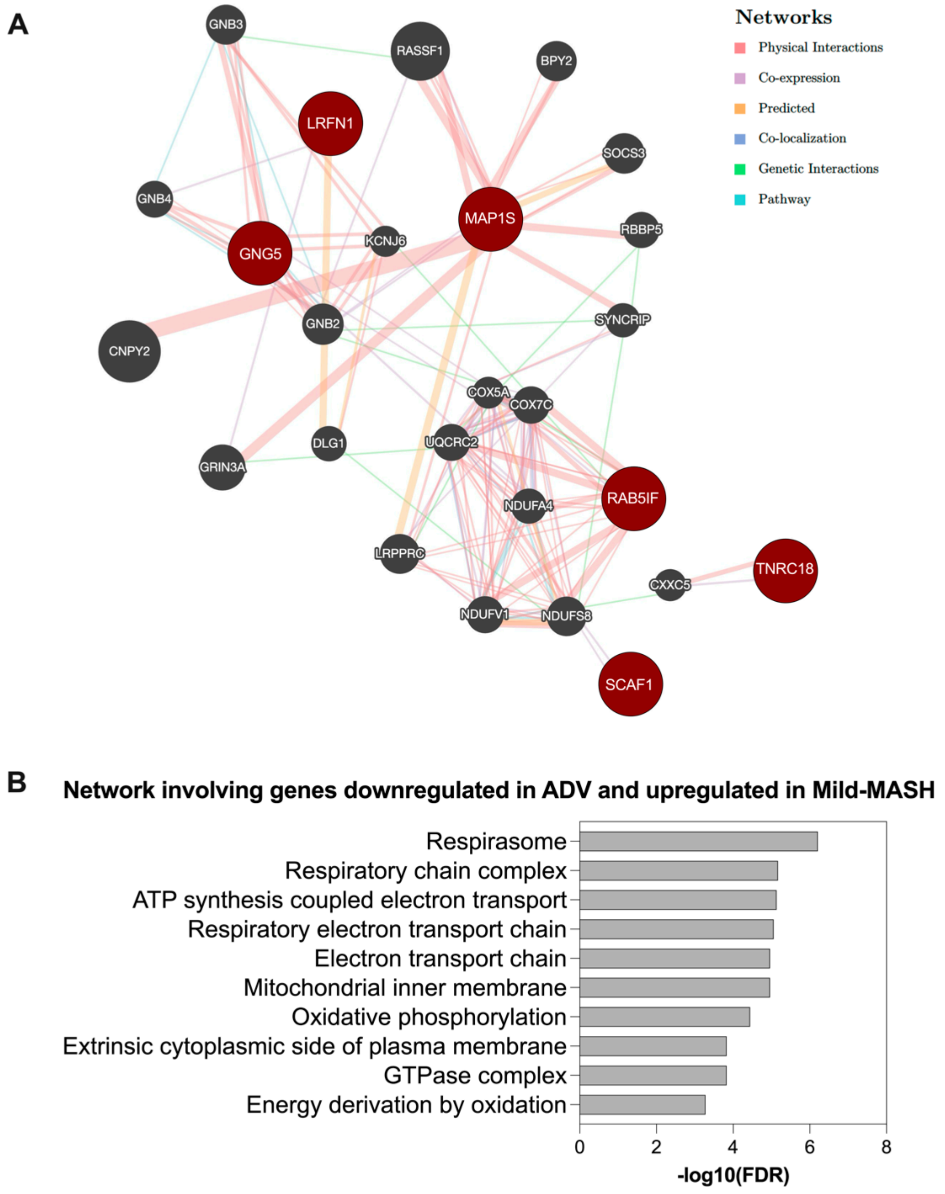

Figure S1. Identification of genes that shift expression in neutrophils from mild to advanced MASH patients. (**A**) Network analysis using GeneMANIA showed that from the 9 genes identified in our dataset, 6 genes are part of a larger network. Legend indicates the type of connection among the genes. (**B**) Pathway enrichment of genes involved in the network from Figure A.

A

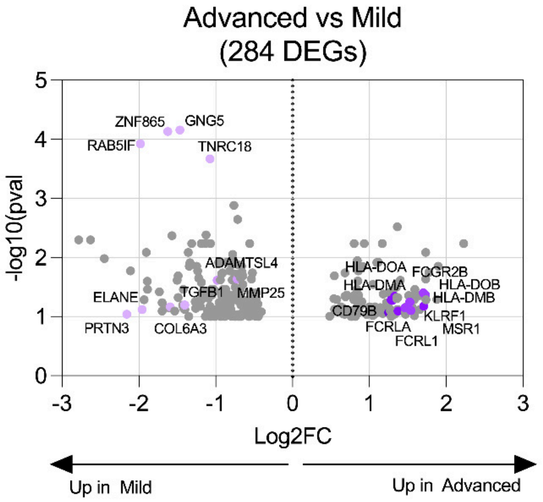

B

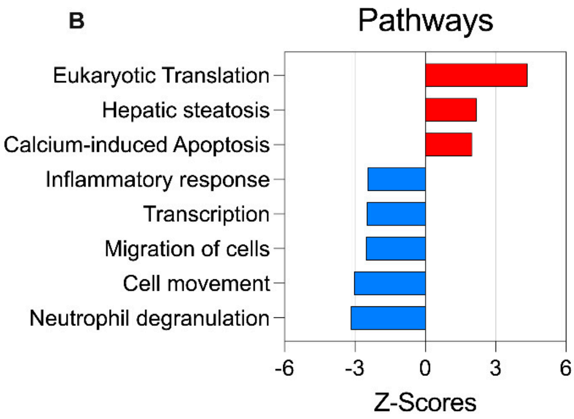

Figure S2. Direct comparison between advanced and mild MASH. (**A**) Volcano plot showing the differentially expressed genes obtained from statistical comparison of advanced and mild transcriptome groups. Arrow to the left: genes upregulated in Mild x Adv. Arrow to the right: genes upregulated in Adv x Mild. (**B**) Pathways enrichment analysis showing pathways upregulated in Mild x Adv (blue) and Adv x Mild (red).
